# Analysis of differences in the rumen microbiome and metabolic function in prepartum dairy cows with different body condition scores

**DOI:** 10.1186/s42523-024-00324-5

**Published:** 2024-06-24

**Authors:** Dewei Du, Yanzhe Wang, Yongji Gao, Lei Feng, Ziye Zhang, Zhiyong Hu

**Affiliations:** Ruminant Nutrition and Physiology Laboratory, College of Animal Science and Technology, Shandong Agricultural University, Tai’an, China

**Keywords:** Prepartum Dairy Cows, Body Condition Score, Rumen Microbiome, Rumen Metabolomics

## Abstract

**Background:**

The rumen is a crucial digestive organ for dairy cows. The rumen microbiota assists in the digestion of plant feed through microbe-mediated fermentation, during which the plant feed is transformed into nutrients for the cow's use. Variations in the composition and function of the rumen microbiome affect the energy utilization efficiency of dairy cows, which is one of the reasons for the varying body condition scores (BCSs). This study focused on prepartum Holstein dairy cows to analyze differences in rumen microbiota and metabolites among cows with different BCSs. Twelve prepartum dairy cows were divided into two groups, low BCS (LBCS, BCS = 2.75, n = 6) and high BCS (HBCS, BCS = 3.5, n = 6), to explore differences in microbial composition and metabolites.

**Results:**

In the HBCS group, the genera within the phylum *Firmicutes* exhibited stronger correlations and greater abundances. Phyla such as *Firmicutes*, *Patescibacteria*, *Acidobacteriota*, *Euryarchaeota*, and *Desulfobacterota*, in addition to most of their constituent microbial groups, were significantly more abundant in the HBCS group than in the LBCS group. At the genus level, the abundances of *Anaerovibrio*, *Veillonellaceae_UCG_001*, *Ruminococcus_gauvreauii_group*, *Blautia*, *Eubacterium*, *Prevotellaceae_YAB2003_group*, *Schwartzia*, and *Halomonas* significantly increased in the HBCS group. The citrate cycle, involved in carbohydrate metabolism, exhibited a significant enrichment trend, with a notable increase in the abundance of its key substrate, citrate, in the HBCS group. This increase was significantly positively correlated with the differential bacterial genera.

**Conclusion:**

In this study, prepartum dairy cows with higher BCS exhibited greater abundance of *Firmicutes*. This study provides theoretical support for microbiological research on dairy cows with different BCSs and suggests that regulating the rumen microbiome could help maintain prepartum dairy cows within an optimal BCS range.

**Supplementary Information:**

The online version contains supplementary material available at 10.1186/s42523-024-00324-5.

## Background

The body condition score (BCS) is a method for assessing the fat reserves and energy metabolism status of dairy cows and represents a crucial area of research for evaluating their physical condition. The tool involves assessing the levels of fat and muscle in cows to monitor animal health and productivity. Studies have highlighted that BCS is vital for the health and care of dairy cows. The scoring system helps monitor nutritional status, thereby preventing health issues caused by undernutrition or overnutrition, which may include metabolic disorders and infections. For instance, the risk of ketosis in cows calving with a BCS > 3.5 is twice as high as that in those with a BCS of 3.25, and cows with a higher BCS are more prone to clinical and subclinical ketosis [1]. Furthermore, the importance of the BCS varies at different stages of a cow's production cycle. Additionally, the BCS is closely related to the production and quality of dairy products. An appropriate BCS can optimize milk yield and quality while reducing the risk of health issues [2]. A study in Irish Holstein–Friesian cattle revealed a significant and sometimes curvilinear relationship between BCS at calving, body weight, and milk yield. For example, cows with a BCS of 4.25 at calving had the highest total milk yield over 305 days. However, cows with BCSs of 3.50, 3.25, and 3.00 produced 68, 118, and 182 kg less milk, respectively, than did those with a BCS of 4.25[3].

The peripartum period is the most critical stage in the production cycle of dairy cows and encompasses 21 days before to 21 days after calving. During the peripartum period, dairy cows undergo complex physiological changes in rumen function, ovarian function, endocrinology, and metabolic status [4]. The BCS during the prepartum period significantly impacts the health and metabolic status of dairy cows. Research by Kabir et al. indicated that the incidences of subclinical hypocalcaemia, hypomagnesemia, hypophosphatemia, and hypoglycaemia vary during the prepartum period. The occurrence of these diseases is related to the BCS and milk production of the cows, with animals having higher BCS and milk yields being more susceptible to these subclinical diseases [5]. Another study focused on the impact of BCS at calving on fat and protein metabolism. This study indicates that cows in the high BCS group exhibited elevated levels of prepartum insulin-like growth factor 1 (IGF-1) and milk fat content, which may be associated with their greater energy reserves and fat mobilization capabilities. Additionally, cows in the high BCS group (BCS ≥ 3.75) produced higher milk fat content and yield compared to the low BCS group, and cows in the low BCS group demonstrated poorer performance in milk protein secretion [6].

The rumen of dairy cows, the first chamber of the stomach, hosts a complex microbial community comprising bacteria, archaea, fungi, and protozoa. These microbes are crucial for cows’ nutrient intake and health and assist in the breakdown and utilization of plant cellulose that cannot be directly digested and absorbed. Volatile fatty acids produced by rumen microbes are a primary energy source for dairy cows, and rumen microbes contribute to the synthesis of vitamins and proteins. Therefore, the health and balance of the rumen microbiome are essential for maintaining the milk production performance and overall health of dairy cows [7, 8]. Changes in the rumen microbial community structure can lead to variations in energy availability in the body, resulting in different body conditions. To date, there has been limited research on the differences in the rumen microbiomes of dairy cows with different BCSs. This study aimed to elucidate the differences in the rumen microbiota among cows with varying BCSs using 16S rRNA gene sequencing and metabolomics approaches. We sought to identify related differentially abundant metabolites, deepening our understanding of the microbial characteristics associated with dairy cow BCS. This will provide a reference for future research.

## Methods

### Animals and samples

**Fig. 1:**
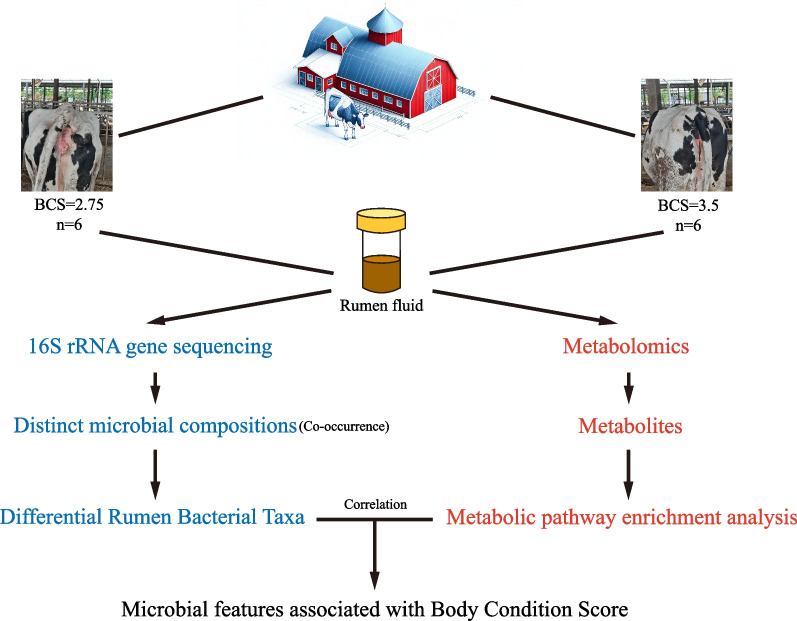
Workflow of the integrated rumen 16S rRNA gene sequencing and metabolomics approaches

The experimental protocol was approved by the Animal Ethics Committee of Shandong Agricultural University (Taian, Shandong). In this experiment, 6 dairy cows with body condition scores (BCSs) of 3.5 and 6 with a BCS of 2.75 were selected. Cows were scored for BCS on a 5-point scale (1–5) with 0.25-unit increments [9]. These cows were all multiparous dairy cows, between their 2nd and 4th lactations, currently in the antepartum period of the periparturient phase. All 12 cows were fed an identical total mixed ration. The total mixed ration included corn silage (45.5%), wheat straw (35.8%) and premix (18.7%). The experiment was conducted 14 days before calving in cows. Rumen content samples were collected using an oral stomach tubing before morning feeding. During sample collection, some of the initially collected fluid was discarded to avoid saliva contamination, and then 50 mL of rumen fluid was collected. Finally, two 2 mL tubes of rumen fluid collected from each cow were preserved. One sample was used for DNA extraction, and the other for metabolomics analysis. The collected samples were immediately preserved in liquid nitrogen and, upon return to the laboratory, were stored in a -80 °C freezer for analysis. The experimental design is illustrated in Fig. 1.

### 16S rRNA gene sequencing and data processing

DNA was extracted from the rumen fluid samples using HiPure Stool DNA Kits, after which the quality of the extracted DNA was checked. Primers (341F: 5′-CCTACGGGNGGCWGCAG-3′; 806R: 5′-GGACTACHVGGGTATCTAAT-3′) were used to amplify the V3-V4 region of the bacterial 16S rRNA gene. The 16S rRNA gene target region were amplified by PCR (95 °C for 5 min, followed by 30 cycles at 95 °C for 1 min, 60 °C for 1 min, and 72 °C for 1 min and a final extension at 72 °C for 7 min) using the aforementioned primers. 50 µL mixture containing 10 µL of 5 × Q5@ Reaction Buffer, 10 µL of 5 × Q5@ High GC Enhancer, 1.5 µL of 2.5 mM dNTPs, 1.5 µL of each primer (10 µM), 0.2 µL of Q5@ High-Fidelity DNA Polymerase, and 50 ng of template DNA. Related PCR reagents were from New England Biolabs, USA. Amplicons were evaluated with 2% agarose gels and purified using the AMPure XP Beads according to the manufacturer’s instructions. Sequencing libraries were generated using Illumina DNA Prep Kit (Illumina, CA, USA) following manufacturer’s recommendation. The library quality was assessed with ABI StepOnePlus Real-Time PCR System (Life Technologies, Foster City, USA). At the end 2 × 250 bp paired-end reads were generated by sequencing on the Novaseq 6000 platform.

After obtaining the raw sequencing data, we first used FASTP software to filter low-quality sequences [10]. The sequences were subsequently assembled using FLASH software [11], in which paired-end sequences were merged into tags. These tags were further filtered to obtain clean tags [12]. Subsequently, the UPARSE algorithm of the USEARCH software [13] was used for clustering based on clean tags, and chimeric tags detected during the clustering comparison process were removed to yield effective tags. After obtaining operational taxonomic units (OTUs), the abundance of the OTUs was calculated based on effective tags. The OTUs were classified into organisms using a naïve Bayesian model with the RDP [14] classifier based on the SILVA database (version 138.1) [15].

The α and β diversities were calculated and visualized using the diversity analysis module of the Omicsmart online analysis platform (http://www.omicsmart.com), and circular stacked diagrams for phyla and genera were created using the species composition analysis module. Spearman correlation analysis was conducted using R software, and significant correlations between different genera were visualized using the R package igraph. Lefse analysis was performed to identify significantly different bacteria between the LBCS and HBCS groups (*P* < 0.05, LDA > 2), and cladogram diagrams were created. GraphPad software was used for the visualization of differentially abundant genera.

### Metabolomics analysis and data processing

The samples were processed using LC–MS/MS to obtain the raw data. These raw data were then converted into MzML format using ProteoWizard, after which peak alignment, retention time correction, and peak area extraction were performed using the XCMS program. The data extracted by XCMS were first checked for completeness. Metabolites with more than 50% missing values within the group were removed and not included in subsequent analyses. Missing values were imputed using the KNN (K-nearest neighbours) method, and outliers were deleted. Finally, the data were normalized to the total peak area.

Partial least squares discriminant analysis (PLS-DA) was performed and visualized using the multivariate statistical analysis module of the Omicsmart online platform (https://www.omicsmart.com). Concurrently, volcano plots were generated using the differential analysis module, and differentially abundant metabolites were identified using T-tests and Variable Importance in Projection (VIP) scores (*P* < 0.05, VIP > 1). These differentially abundant metabolites were then subjected to Kyoto Encyclopedia of Genes and Genomes (KEGG) enrichment analysis. The R packages ggplot2 and ggsankey were utilized to visualize the results, creating Sankey bubble combination diagrams. Additionally, the R package pheatmap was used to calculate and construct a Spearman correlation heatmap.

## Results

### Associations between the rumen microbiota and body condition scores

**Fig. 2:**
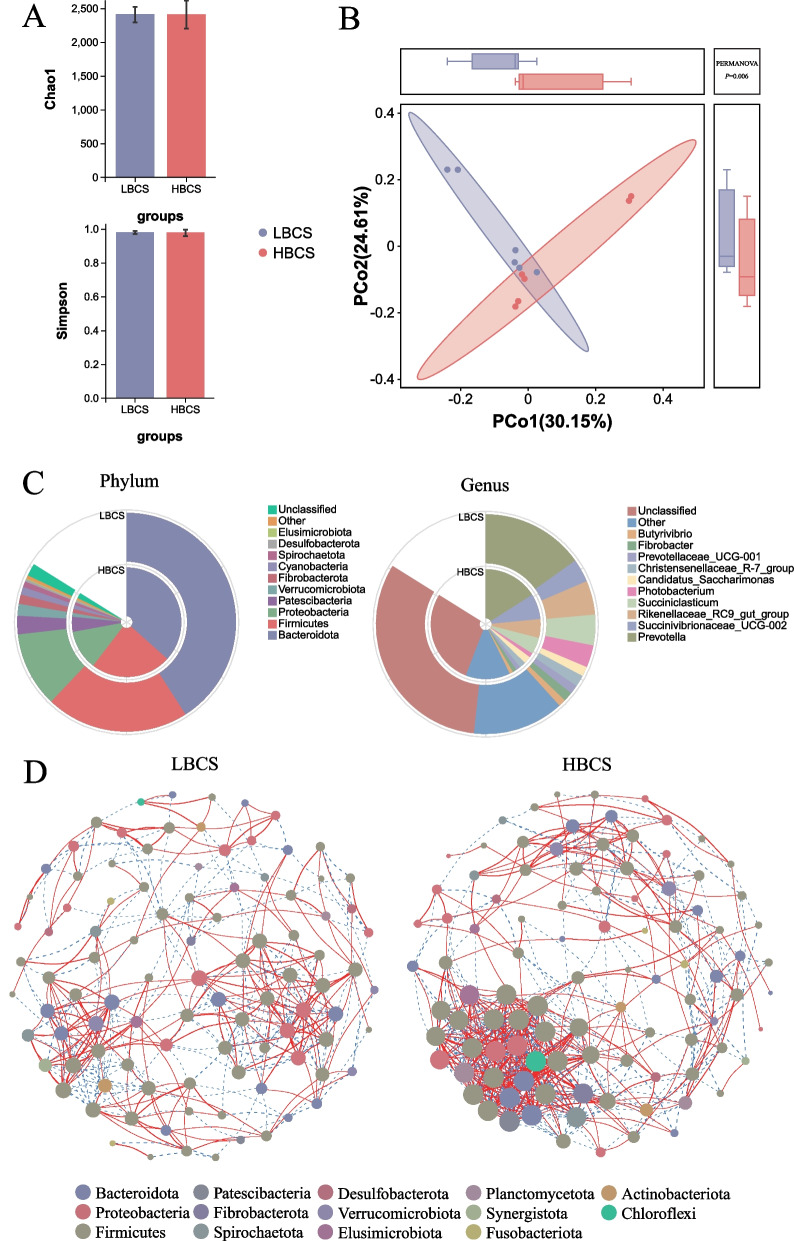
The difference in the rumen microbiome between cows with low (LBCS) and high (HBCS) body condition scores according to 16S rRNA gene sequencing data. **A** Changes in alpha diversity at the OTU level. **B** Changes in beta diversity at the OTU level. The *P* value was tested with PERMANOVA. **C** Community biplot analysis at the phylum and genus levels. **D** Genus co-occurrence network between LBCS and HBCS based on Spearman correlation analysis. Each node represents a bacterial genus; the node size represents the relative abundance of each genus per group. The line refers to the Spearman coefficient. Red and blue lines represent positive and negative interactions between nodes, respectively

16S rRNA gene sequencing generated a total of 1,514,409 raw reads, with an average of 126,201 reads per sample. After filtering, merging, and clustering through USEARCH software and removing chimaeras, OTU sequences were obtained, averaging 2344 OTUs per sample. α-Diversity analysis at the OTU level revealed no significant differences between the LBCS and HBCS groups in terms of the Chao1 and Simpson indices (*P* > 0.05) (Fig. 2A). However, β diversity analysis based on Bray–Curtis distance indicated significant differences between the two groups. This was confirmed by permutational multivariate analysis of variance (PERMANOVA), which revealed significant differences in the rumen microbiota structure of prepartum dairy cows with different body condition scores (*P* = 0.006) (Fig. 2B). Annotation was performed using the SILVA database, focusing on the top 10 phyla and genera. At the phylum level, *Bacteroidota*, *Firmicutes*, and *Proteobacteria* were the dominant phyla in the rumen, followed by *Patescibacteria*, *Verrucomicrobiota*, *Fibrobacterota*, *Cyanobacteria*, *Spirochaetota*, *Desulfobacterota*, and *Elusimicrobiota*. At the genus level, *Prevotella*, *Succinivibrionaceae_UCG-002*, *Rikenellaceae_RC9_gut_group*, *Succiniclasticum*, *Photobacterium*, *Candidatus_Saccharimonas*, *Christensenellaceae_R-7_group*, *Prevotellaceae_UCG-001*, *Fibrobacter*, and *Butyrivibrio* were identified as the core microbiota in the rumens of both groups (Fig. 2C). Co-occurrence network analysis at the genus level revealed 391 co-linearity relationships in the LBCS group and 629 in the HBCS group, with the LBCS group exhibiting notably fewer edges. In both groups, the phylum *Firmicutes* had the most genera, but the microbial structures were not the same between the groups. In the HBCS group, the abundance of genera within the *Firmicutes* phylum was greater than that in the other groups (Fig. 2D).

**Fig. 3:**
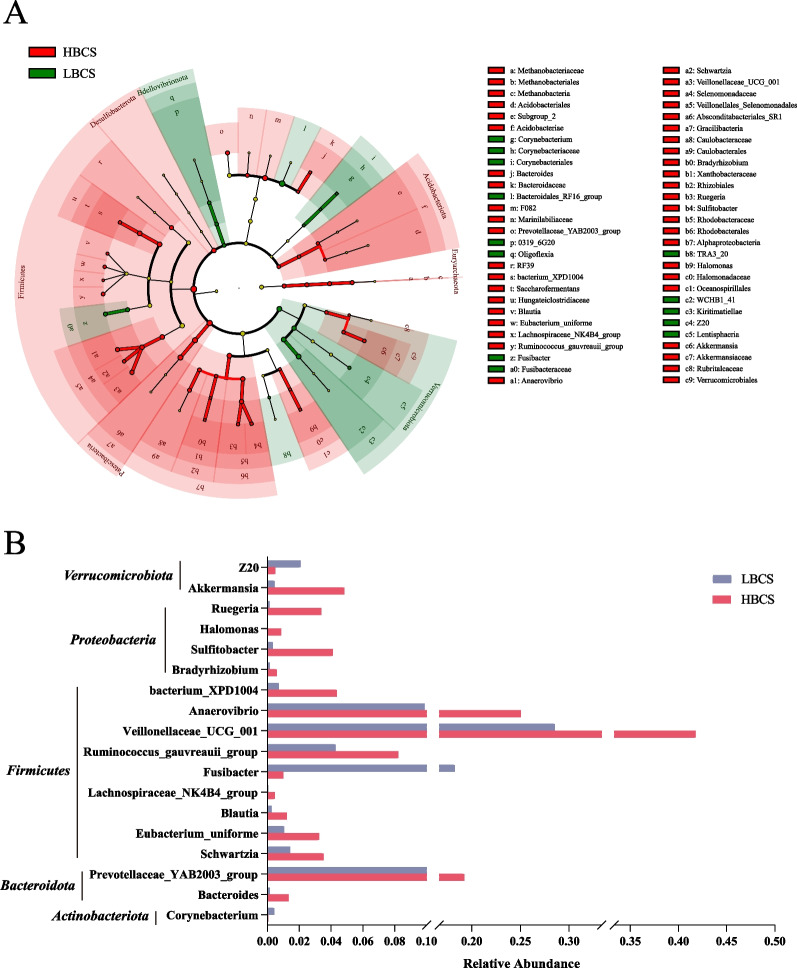
The difference in the rumen microbiota between cows with low (LBCS) and high (HBCS) body condition scores according to 16S rRNA gene sequencing data. **A** Cladogram of significantly different rumen microbiota between LBCS and HBCS. Significant differences were tested by linear discriminant analysis effect size analysis, with linear discriminant analysis (LDA) scores > 2 and a *P* value < 0.05. **B** Abundance of significantly different bacterial genera between LBCS and HBCS. Significant differences were tested by linear discriminant analysis effect size analysis, with linear discriminant analysis (LDA) scores > 2 and a *P* value < 0.05

Lefse analysis revealed a total of 63 differential bacterial taxa, ranging from phylum to genus, between the two groups (*P* < 0.05, LDA > 2). Within the Firmicutes phylum, all taxa except for Fusibacteraceae and Fusibacter were more abundant in the HBCS group than in the LBCS group. The phyla *Patescibacteria*, *Acidobacteriota*, *Euryarchaeota*, and *Desulfobacterota*, along with their constituent taxa, exhibited significantly greater abundances in the HBCS group. Within the *Verrucomicrobiota* phylum, except for *Akkermansia*, *Akkermansiaceae*, *Rubritaleaceae*, and *Verrucomicrobiales*, the remaining taxa were more abundant in the LBCS group. Additionally, the abundances of *Bdellovibrionota*, *0319_6G20*, and *Oligoflexia* were significantly greater in the LBCS group (Fig. 3A). Through Lefse analysis, 18 differential genera were identified (*P* < 0.05, LDA > 2), with the majority belonging to the *Firmicutes* phylum. Except for *Fusibacter*, *Z20*, and *Corynebacterium*, all other genera were significantly elevated in the HBCS group (Fig. 3B).

### Associations between rumen metabolites and body condition scores

**Fig. 4:**
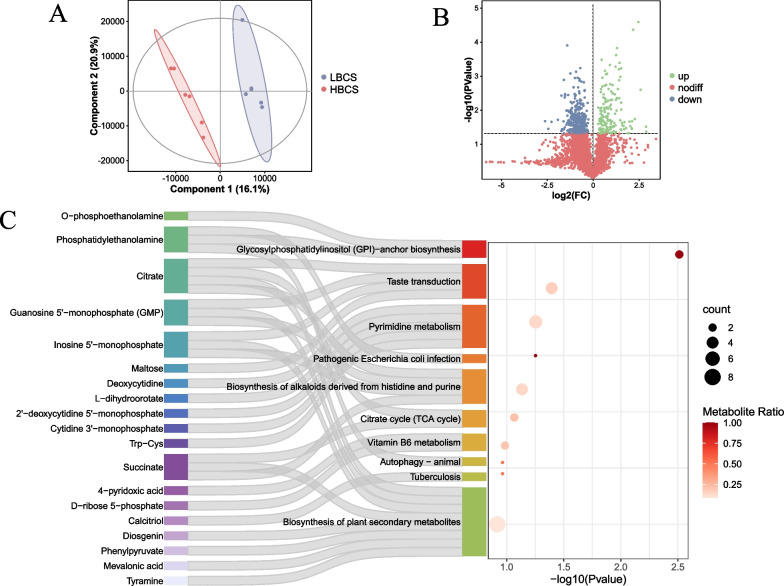
Metabolic pathway enrichment analysis of differential rumen metabolites between cows with low (LBCS) and high body condition scores. **A** partial least squares-discriminant analysis of the rumen metabolome between LBCS and HBCS cows. **B** Volcano map of metabolites identified in the faecal metabolome. **C** Pathway enrichment analysis of metabolites

Nontargeted metabolomics analysis was conducted on the rumen fluid of the LBCS and HBCS cows. The partial least squares discriminant analysis (PLS-DA) results revealed distinct clustering between the metabolites of the two groups (Fig. 4A). A total of 5138 metabolites were detected and quantified, among which 217 were identified as differentially abundant metabolites (*P* < 0.05, VIP > 1) (Table S1). Specifically, in the HBCS group, the abundance of 77 metabolites was significantly greater than that in the LBCS group, while the abundance of 140 metabolites was significantly lower (Fig. 4B). Subsequently, we conducted differential KEGG pathway enrichment analysis on these differentially abundant metabolites and focused on the top 10 pathways with significant differences, encompassing 19 differentially abundant metabolites (Fig. 4C). The results showed significant enrichment in glycosylphosphatidylinositol (GPI)-anchor biosynthesis (*P* = 0.003) and taste transduction pathways (*P* = 0.04), indicating significant differences between HBCS and LBCS cows. Additionally, pyrimidine metabolism, pathogenic *Escherichia coli* infection, biosynthesis of alkaloids derived from histidine and purine, and the citrate cycle (TCA cycle) showed trends towards differences (0.05 < *P* < 0.1).

These 19 differentially abundant metabolites were categorized into different classes: organic acids and derivatives included Trp-Cys, O-phosphoethanolamine, succinate, L-dihydroorotate, and citrate; benzenoids included Tyramine; lipids and lipid-like molecules included phosphatidylethanolamine, mevalonic acid, diosgenin, and calcitriol; organic oxygen compounds included maltose, D-ribose 5-phosphate, and cytidine 3'-monophosphate; nucleosides, nucleotides, and analogues included inosine 5'-monophosphate, deoxycytidine, and 2'-deoxycytidine 5'-monophosphate; and organoheterocyclic compounds included 4-pyridoxic acid.

**Fig. 5:**
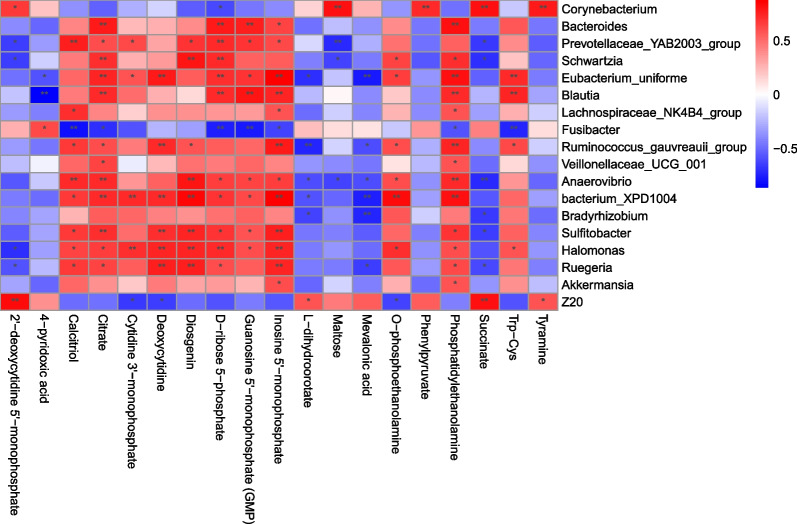
Correlation analysis of the significantly differential microbes and metabolites. Spearman correlations between the significantly differential microbiota and metabolites. *Represents a correlation *P* value < 0.05, **P value < 0.01. Red and blue represent positive and negative correlations, respectively

Correlation analysis indicated a significant association (*P* < 0.05) between the bacterial genera with significant differences and these differentially abundant metabolites (Fig. 5). O-phosphoethanolamine, phosphatidylethanolamine, Trp-Cys, calcitriol, citrate, cytidine 3'-monophosphate, deoxycytidine, diosgenin, D-ribose 5-phosphate, guanosine 5'-monophosphate (GMP), and inosine 5'-monophosphate showed the most significant positive correlations with the bacterial populations that were significantly enriched in the HBCS group.

## Discussion

Maintaining an appropriate BCS in dairy cows during the peripartum period is beneficial for the health of the cows postpartum and positively impacts their productive performance. Current research indicates that there is a certain correlation between the feed efficiency, methane emissions, and lactation performance of dairy cows and the rumen microbiome [16–18]. In our study, although there were no significant differences in α diversity, β diversity analysis revealed distinct differences in the microbial community structure between the LBCS and HBCS groups. Subsequent co-occurrence network analysis revealed that, in the HBCS group, the abundance and correlation of bacterial genera in the phylum *Firmicutes* in the rumen were greater. Bacteria within *Firmicutes* in the rumen play a crucial role in the health and productive performance of dairy cows. *Firmicutes* are key to the breakdown of cellulose and other hard-to-digest substances in the rumen and are vital for extracting energy from roughage in dairy cows [19]. Research indicates that *Firmicutes* is one of the most important bacterial groups in the rumen, fermenting plant fibres to produce volatile fatty acids (VFAs), which are a major energy source for dairy cows [20]. In human gut microbiome studies, researchers have shown that, compared to individuals of normal weight, obese animals and humans exhibit a higher ratio of *Firmicutes*/*Bacteroidetes* in their gut microbiota. *Firmicutes* species are more efficient at extracting energy from food, thus promoting more effective caloric absorption and subsequent weight gain [21]. This may be associated with the extraction of more energy from the diet leading to obesity. The differences of BCS observed during the dry period originate from the previous mid and late lactation, and they are largely due to genetically driven differences in nutrient partitioning between body tissues and lactating mammary gland [22]. In the HBCS group, the genera within the phylum *Firmicutes* exhibited stronger correlations and greater abundances, which could be related to cows in higher body condition score groups extracting more energy from the same diet, potentially leading to obesity [23].

In our study, the cladogram shows that the abundance of most differential bacterial groups significantly increased in the HBCS group. These groups were concentrated in the phyla *Firmicutes*, *Patescibacteria*, *Acidobacteriota*, *Euryarchaeota*, and *Desulfobacterota*. *Patescibacteria* are a relatively newly discovered class of bacteria. They are characterized by their small size and correspondingly reduced genomes. These bacteria are generally considered to rely on symbiotic relationships with other microorganisms to supplement their metabolic pathways [24]. Studies have indicated that in the gut of mice, there is a significant positive correlation between *Patescobacteria* and short-chain fatty acids (SCFAs) [25]. Furthermore, in a study by Wang et al., a comparison of the differential bacterial communities in the rumens of hoofed animals at high and low altitudes revealed that *Patescibacteria* might reduce energy loss in hoofed animals in high-altitude environments [26]. This finding suggested that dairy cows with higher body condition scores may better utilize volatile fatty acids through *Patescibacteria*, thereby providing energy to the organism. *Euryarchaeota* are a class of archaea that encompasses most species within archaea and often includes methanogens found in animal intestines. In the cladogram, the abundances of *Methanobacteria*, *Methanobacteriales*, and *Methanobacteriaceae* significantly increased in the HBCS group, all of which are associated with methane production [27]. The primary function of these archaea is to generate methane using substrates obtained during rumen fermentation, an interesting phenomenon as increased methane production signifies a reduction in energy utilization efficiency, which leads to decreased energy for fat accumulation. This issue will require further investigation in the future. The phyla *Acidobacteriota* and *Desulfobacterota* have received less attention in dairy cow research, necessitating further studies to confirm their roles in energy utilization efficiency.

Among the differential bacterial genera between the HBCS and LBCS groups, most belonged to the phylum *Firmicutes*. Research on the genus *Anaerovibrio* in the rumen has focused primarily on its role in fat metabolism [28, 29]. In particular, *Anaerovibrio lipolyticus* is considered one of the main lipolytic bacteria in the rumen and is capable of removing fat from the rumen for improved absorption and utilization, thereby enhancing the body's efficiency in fat utilization. *Veillonellaceae_UCG_001* and *Ruminococcus_gauvreauii_group* are bacteria belonging to the phylum *Firmicutes*. In a study on the rumen microbiota of yaks, researchers found that supplementation with isoacids could improve fibre digestibility, mainly through increasing cellulase activity and the number of cellulose-producing bacteria in the rumen. The abundances of *Veillonellaceae_UCG-001* and *Ruminococcus_gauvreauii_group* significantly increased after the supplementation of isoacids [30].

*Blautia* is a genus of anaerobic bacteria with probiotic properties that are widely found in the faeces and intestines of mammals and are primarily known for their biotransformation abilities and roles in regulating host health and alleviating metabolic syndrome [31]. *Eubacterium*, one of the dominant genera in the gut microbiota [32], produces a mixture of organic acids from carbohydrates or peptone, including substantial amounts of butyrate, acetate, and formate, thus enhancing the body's carbohydrate utilization. With respect to *Prevotellaceae_YAB2003_group* and *Schwartzia*, research has investigated the impact of artificial saliva with varying pH levels on rumen fermentation and bacterial communities. A lower artificial saliva pH reduced the degradability of dry matter, organic matter, neutral detergent fibre, and acid detergent fibre and decreased the abundance of *Prevotellaceae_YAB2003_group* and *Schwartzia* [33], suggesting their potential role in fibre degradation. Studies on *Halomonas* have focused primarily on its role in marine environments, but research by Xue et al. suggested that a decrease in *Halomonas* abundance may indicate decreased nitrogen transformation and utilization efficiency [34]. In summary, dairy cows with higher body condition scores have a greater abundance of rumen bacterial genera related to cellulose decomposition and short-chain fatty acid production, which facilitates better utilization of plant fibres and enhances the production of volatile fatty acids. This could be a contributing factor to the higher BCSs in these cows.

Metabolomics plays a crucial role in the study of the rumen in dairy cows. This study analysed the rumen metabolome to understand the differences in metabolites in cows with varying body condition scores, thereby inferring the metabolic status of the rumen microbiome. The study revealed that among the 19 differentially abundant metabolites enriched in the top 10 pathways, the majority were organic acids and derivatives and lipids and lipid-like molecules. Notably, a significant enrichment trend was observed for the citrate cycle, a carbohydrate metabolism pathway. Citrate was significantly positively correlated with most bacterial genera that were abundant in the HBCS group. Research has shown that feeding cows silage treated with lignocellulose-degrading bacteria enhances the functional characteristics of the citrate cycle in the rumen, thereby improving the digestion rate of neutral detergent fiber (NDF) [35]. Packett and Fordham studied the utilization of citrate by sheep rumen microbiota and reported that citrate was rapidly used by rumen microbes to increase VFA production and the percentage of acetate [36]. Furthermore, Wright's study also indicated that rumen microbes quickly convert citrate to carbon dioxide and acetate, with the metabolic rate increasing as the concentration of citrate increases [37]. In summary, certain microbial groups in the rumens of HBCS cows have a stronger citrate cycle pathway, thereby more effectively enhancing the production of volatile fatty acids.

This study identified differences in the microbial communities and metabolites in the rumens of periparturient dairy cows with varying BCSs. However, using only 16S rRNA gene sequencing and metabolomics cannot fully elucidate the mechanisms underlying differences in BCSs. In future research, we plan to measure volatile fatty acids from the rumen, as well as measure the production performance and investigate the blood indicators identified in this study. We will integrate these methods with multiomics analysis methods, including metagenomics and metatranscriptomics, to provide more robust evidence for these biological mechanisms.

## Conclusions

In summary, in the HBCS group, there was a significant increase in the abundance of Firmicutes bacteria. Additionally, due to changes in the rumen microbial community structure, the metabolites in the rumen also changed, with citrate, which participates in the citric acid cycle, being more abundant in the HBCS group. These results have directed our future research efforts and laid the foundation for the development and utilization of probiotics.

### Supplementary Information

**Additional file 1**.

## Data Availability

The Illumina sequencing raw data for our samples have been deposited in the NCBI Sequence Read Archive (SRA) under accession numbers: PRJNA1055746.

## Abbreviations

BCS: Body condition score
HBCS: High body condition score
LBCS: Low body condition score
PLS-DA: Partial least squares discriminant analysis
KEGG: Kyoto Encyclopedia of Genes and Genomes
PERMANOVA: Permutational multivariate analysis of variance
LDA: Linear discriminant analysis
VFAs: Volatile fatty acids
SCFAs: Short-chain fatty acids
NDF: Neutral detergent fiber

## References

[1] Gillund P, Reksen O, Gröhn YT, Karlberg K (2001). Body condition related to ketosis and reproductive performance in Norwegian dairy cows. J Dairy Sci.

[2] Roche JR, Friggens NC, Kay JK, Fisher MW, Stafford KJ, Berry DP (2009). Invited review: body condition score and its association with dairy cow productivity, health, and welfare. J Dairy Sci.

[3] Berry DP, Buckley F, Dillon P (2007). Body condition score and live-weight effects on milk production in Irish Holstein-Friesian dairy cows. Animal.

[4] Rathbun FM, Pralle RS, Bertics SJ, Armentano LE, Cho K, Do C (2017). Relationships between body condition score change, prior mid-lactation phenotypic residual feed intake, and hyperketonemia onset in transition dairy cows. J Dairy Sci.

[5] Kabir M, Hasan MM, Tanni NS, Parvin MS, Asaduzzaman M, Ehsan MA (2022). Metabolic profiling in periparturient dairy cows and its relation with metabolic diseases. BMC Res Notes.

[6] Pires JA, Delavaud C, Faulconnier Y, Pomiès D, Chilliard Y (2013). Effects of body condition score at calving on indicators of fat and protein mobilization of periparturient Holstein–Friesian cows. J Dairy Sci.

[7] Xu Q, Qiao Q, Gao Y, Hou J, Hu M, Du Y (2021). Gut microbiota and their role in health and metabolic disease of dairy cow. Front Nutr.

[8] Huang S, Ji S, Suen G, Wang F, Li S (2021). The rumen bacterial community in dairy cows is correlated to production traits during freshening period. Front Microbiol.

[9] Ferguson JD, Galligan DT, Thomsen N (1994). Principal descriptors of body condition score in Holstein cows. J Dairy Sci.

[10] Chen S, Zhou Y, Chen Y, Gu J (2018). fastp: an ultra-fast all-in-one FASTQ preprocessor. Bioinformatics.

[11] Magoč T, Salzberg SL (2011). FLASH: fast length adjustment of short reads to improve genome assemblies. Bioinformatics.

[12] Bokulich NA, Subramanian S, Faith JJ, Gevers D, Gordon JI, Knight R (2013). Quality-filtering vastly improves diversity estimates from Illumina amplicon sequencing. Nat Methods.

[13] Edgar RC (2013). UPARSE: highly accurate OTU sequences from microbial amplicon reads. Nat Methods.

[14] Wang Q, Garrity GM, Tiedje JM, Cole JR (2007). Naive Bayesian classifier for rapid assignment of rRNA sequences into the new bacterial taxonomy. Appl Environ Microbiol.

[15] Pruesse E, Quast C, Knittel K, Fuchs BM, Ludwig W, Peplies J (2007). SILVA: a comprehensive online resource for quality checked and aligned ribosomal RNA sequence data compatible with ARB. Nucleic Acids Res.

[16] Xue MY, Xie YY, Zhong Y, Ma XJ, Sun HZ, Liu JX (2022). Integrated meta-omics reveals new ruminal microbial features associated with feed efficiency in dairy cattle. Microbiome.

[17] Shi W, Moon CD, Leahy SC, Kang D, Froula J, Kittelmann S (2014). Methane yield phenotypes linked to differential gene expression in the sheep rumen microbiome. Genome Res.

[18] Jami E, Mizrahi I (2012). Composition and similarity of bovine rumen microbiota across individual animals. PLoS ONE.

[19] Liu L, Wu P, Guo A, Yang Y, Chen F, Zhang Q (2023). Research progress on the regulation of production traits by gastrointestinal microbiota in dairy cows. Front Vet Sci.

[20] Mizrahi I, Wallace RJ, Moraïs S (2021). The rumen microbiome: balancing food security and environmental impacts. Nat Rev Microbiol.

[21] Magne F, Gotteland M, Gauthier L, Zazueta A, Pesoa S, Navarrete P, et al. The firmicutes/bacteroidetes ratio: a relevant marker of gut dysbiosis in obese patients? Nutrients. 2020;12(5). 10.3390/nu12051474 PMC7285218 32438689

[22] Friggens NC, Brun-Lafleur L, Faverdin P, Sauvant D, Martin O (2013). Advances in predicting nutrient partitioning in the dairy cow: recognizing the central role of genotype and its expression through time. Animal.

[23] Krajmalnik-Brown R, Ilhan ZE, Kang DW, DiBaise JK (2012). Effects of gut microbes on nutrient absorption and energy regulation. Nutr Clin Pract.

[24] Wang Y, Gallagher LA, Andrade PA, Liu A, Humphreys IR, Turkarslan S (2023). Genetic manipulation of Patescibacteria provides mechanistic insights into microbial dark matter and the epibiotic lifestyle. Cell.

[25] Hao R, Zhou X, Zhao X, Lv X, Zhu X, Gao N (2023). Flammulina velutipes polysaccharide counteracts cadmium-induced gut injury in mice via modulating gut inflammation, gut microbiota and intestinal barrier. Sci Total Environ.

[26] Wang X, Wu X, Shang Y, Gao Y, Li Y, Wei Q (2022). High-altitude drives the convergent evolution of alpha diversity and indicator microbiota in the gut microbiomes of ungulates. Front Microbiol.

[27] Malik PK, Trivedi S, Kolte AP, Mohapatra A, Biswas S, Bhattar AVK (2023). Comparative analysis of rumen metagenome, metatranscriptome, fermentation and methane yield in cattle and buffaloes fed on the same diet. Front Microbiol.

[28] Prins RA, Lankhorst A, van der Meer P, Van Nevel CJ (1975). Some characteristics of Anaerovibrio lipolytica a rumen lipolytic organism. Antonie Van Leeuwenhoek.

[29] Henderson C (1970). The lipases produced by Anaerovibrio lipolytica in continuous culture. Biochem J.

[30] Jiang F, Gao Y, Peng Z, Ma X, You Y, Hu Z (2023). Isoacids supplementation improves growth performance and feed fiber digestibility associated with ruminal bacterial community in yaks. Front Microbiol.

[31] Liu X, Mao B, Gu J, Wu J, Cui S, Wang G (2021). Blautia: a new functional genus with potential probiotic properties?. Gut Microbes.

[32] Li WD, Li LS, Lyu MJ, Hu QY, Xiong DQ (2023). Research progress of Eubacterium and its metabolite short-chain fatty acids in regulating type 2 diabetes mellitus. Zhonghua Yu Fang Yi Xue Za Zhi.

[33] Guo T, Guo T, Cao Y, Guo L, Li F, Li F (2021). Changes in the fermentation and bacterial community by artificial saliva pH in RUSITEC system. Front Nutr.

[34] Xue Y, Lin L, Hu F, Zhu W, Mao S (2020). Disruption of ruminal homeostasis by malnutrition involved in systemic ruminal microbiota-host interactions in a pregnant sheep model. Microbiome.

[35] Guo W, Guo XJ, Xu LN, Shao LW, Zhu BC, Liu H (2022). Effect of whole-plant corn silage treated with lignocellulose-degrading bacteria on growth performance, rumen fermentation, and rumen microflora in sheep. Animal.

[36] Packett LV, Fordham JR (1965). Utilization of citric acid by rumen microorganisms. J Anim Sci.

[37] Wright DE (1971). Citric acid metabolism in the bovine rumen. Appl Microbiol.

